# Cases of Fracture and Dislocation, Reported to the Medical Society of Tennessee, in May, 1841

**Published:** 1841-08

**Authors:** Felix Robertson

**Affiliations:** Nashville, Tenn.


ART. IV.—*Cases of Fracture and Dislocation*, reported to the Medical Society of Tennessee, in May, 1841. By FELIX ROBERTSON, M. D., of Nashville, Tenn.

*Case 1st. Dislocation of the Neck.*—Many years ago, I was called ten miles into the country to see James Stuart, an Irishman, beyond the middle term of life. I reached the house where he lay about sunrise, and learnt that about sunset, the evening before, in attempting to mount his horse, while in a state of intoxication, he had gone over the animal and fallen, head foremost, on the ground. The fall occurred on quite a steep hill side, and he fell down the hill. Two or three men were in company with him, and were much amused to see him throw such a somerset, but, although he remained perfectly motionless, did not go to him for some minutes. When one did so, and attempted to raise him up, the man was found to be apparently dead. He was immediately laid on his back and straightened out, when it was soon discovered that he breathed, and he continued gradually to recover until he was able to speak in a whisper; but from his head down, it was ascertained that he was completely paralysed, and that all sensibility was gone.

When I saw him, in the morning, he complained of nothing, could converse in a whisper with his usual intelligence, but pins thrust into any part of his body or limbs gave not the slightest pain, and all muscular motion was impracticable. Conceiving that a partial dislocation of some of the cervical vertebræ existed, and that the case justified a resort to the most vigorous remedies, I placed a man at each of his legs, and after passing a saddle girt under his chin and secured it so that it could not slip off, gave the ends to two other assistants. After several trials, in which I began at last seriously to fear that his head would be severed from his body, I had the satisfaction to succeed in reducing the dislocation. The parts returned to their proper situation with a snap, that was distinctly heard by all persons around, leaving no room to doubt, that a dislocation had existed.

In a few minutes, after the reduction, the patient complained of a singular sensation of heat in all parts of his body, such as he wnuld feel if standing near the fire; but still, for a number of days, he was insensible to the prick of a pin. Sensibility did, however, very gradually return, until it acquired nearly its natural tone, and with it, the power of muscular motion; but the latter function was established much more slowly than the former.

I saw him some months after the accident, when he was in the following condition: general health good, except constipation of the bowels, which had persisted since the time of his fall; he was able to sit on his chair, and to move his arms and legs, but was not able to walk, or to use his knife and fork. He was sanguine about a complete recovery.

Three months subsequently he was attacked with bilious colic, and failing, as he lived far in the country, to procure medical aid, he sunk under the disease on the second day of the attack. No post mortem examination was made, which is much to be regretted, since it might have disclosed the extent of the local injury that had been sustained in the fall.

*Case 2nd. Compound Fracture of the Arm at the Elbow Joint.*—A messenger came to me from Mrs. A.'s, four miles in the country, with a request that I would meet my partner, Dr. Waters, taking with me my instruments for amputating the arm. I learnt from the messenger that the son of Mrs. A. had fallen from a tree, and had broken his arm so badly, that Dr. Waters deemed an amputation necessary. On examining the arm of the lad, aged 8 or 9 years, I found that the humerus had been thrown forward, breaking up all the soft parts in front, and passing entirely through, so as to be exposed, to the extent of several inches. In its violent passage, in fact, it seemed to have nearly cut off the arm, and the soft parts closing behind it, there appeared but little prospect of our being able to restore it to its proper position.

It was determined, however, to attempt to save the limb, and accordingly the ivory handle of a scalpel was passed into the wound, on each side of the bone, which acted as smooth surfaces on which the bone might slide, and at the same time the sides of the wound were pressed asunder. By this method the fractured bone was returned to its place, and it was surprising to see what a change was thus effected in the appearance of the wound, which, now, closing over the bone left but little resemblance of what it had been a few minutes before. The proper inclination was given to the arm, and that position secured by a bandage. A speedy recovery followed, of all but the use of the elbow joint. Except this inevitable anchylosis, the arm was restored to perfect usefulness.

This case is reported to the Society rather on account of its rare occurrence, than from any idea that it presented any thing novel or peculiar.

*Nashville, May* 3, 1841.

